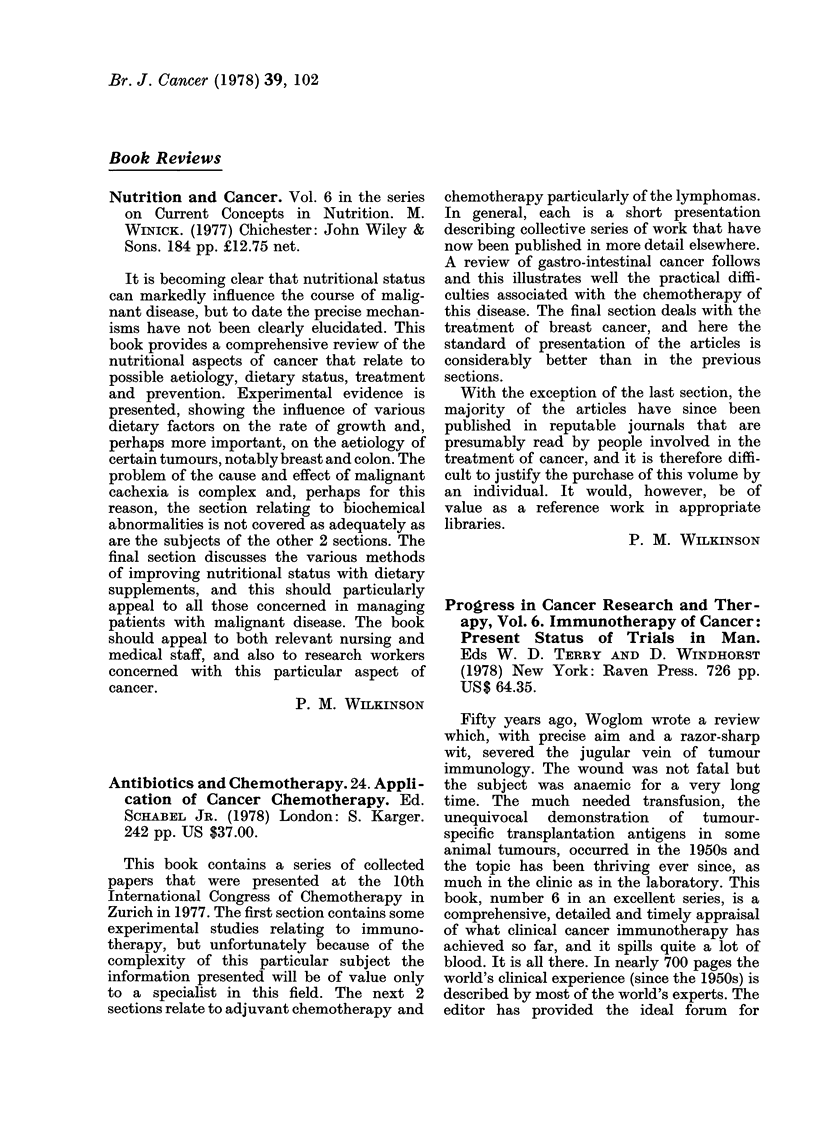# Nutrition and Cancer

**Published:** 1979-01

**Authors:** P. M. Wilkinson


### *Book Reviews*

**Nutrition and Cancer.** Vol. 6 in the series on Current Concepts in Nutrition. M. WINICK. (1977) Chichester: John Wiley & Sons. 184 pp. £12.75 net.

It is becoming clear that nutritional status can markedly influence the course of malignant disease, but to date the precise mechanisms have not been clearly elucidated. This book provides a comprehensive review of the nutritional aspects of cancer that relate to possible aetiology, dietary status, treatment and prevention. Experimental evidence is presented, showing the influence of various dietary factors on the rate of growth and, perhaps more important, on the aetiology of certain tumours, notably breast and colon. The problem of the cause and effect of malignant cachexia is complex and, perhaps for this reason, the section relating to biochemical abnormalities is not covered as adequately as are the subjects of the other 2 sections. The final section discusses the various methods of improving nutritional status with dietary supplements, and this should particularly appeal to all those concerned in managing patients with malignant disease. The book should appeal to both relevant nursing and medical staff, and also to research workers concerned with this particular aspect of cancer.

P. M. WILKINSON